# Does it pay to pay? A comparison of the benefits of open-access publishing across various sub-fields in biology

**DOI:** 10.7717/peerj.16824

**Published:** 2024-02-27

**Authors:** Amanda D. Clark, Tanner C. Myers, Todd D. Steury, Ali Krzton, Julio Yanes, Angela Barber, Jacqueline Barry, Subarna Barua, Katherine Eaton, Devadatta Gosavi, Rebecca Nance, Zahida Pervaiz, Chidozie Ugochukwu, Patricia Hartman, Laurie S. Stevison

**Affiliations:** 1 Department of Biological Sciences, Auburn University, Auburn, AL, United States of America; 2 Department of Cell, Developmental, and Integrative Biology, University of Alabama-Birmingham, Birmingham, AL, United States of America; 3 College of Forestry, Wildlife, and Environment, Auburn University, Auburn, AL, United States of America; 4 Auburn University Libraries, Auburn University, Auburn, AL, United States of America; 5 Department of Pathobiology, Auburn University, Auburn, AL, United States of America; 6 Department of Chemistry and Biochemistry, Auburn University, Auburn, AL, United States of America

**Keywords:** Open-access publishing, Paywall, Hybrid journal, Article processing charge, Citation advantage, Mixed-effect model

## Abstract

Authors are often faced with the decision of whether to maximize traditional impact metrics or minimize costs when choosing where to publish the results of their research. Many subscription-based journals now offer the option of paying an article processing charge (APC) to make their work open. Though such “hybrid” journals make research more accessible to readers, their APCs often come with high price tags and can exclude authors who lack the capacity to pay to make their research accessible. Here, we tested if paying to publish open access in a subscription-based journal benefited authors by conferring more citations relative to closed access articles. We identified 146,415 articles published in 152 hybrid journals in the field of biology from 2013–2018 to compare the number of citations between various types of open access and closed access articles. In a simple generalized linear model analysis of our full dataset, we found that publishing open access in hybrid journals that offer the option confers an average citation advantage to authors of 17.8 citations compared to closed access articles in similar journals. After taking into account the number of authors, Journal Citation Reports 2020 Quartile, year of publication, and Web of Science category, we still found that open access generated significantly more citations than closed access (*p* < 0.0001). However, results were complex, with exact differences in citation rates among access types impacted by these other variables. This citation advantage based on access type was even similar when comparing open and closed access articles published in the same issue of a journal (*p* < 0.0001). However, by examining articles where the authors paid an article processing charge, we found that cost itself was not predictive of citation rates (*p* = 0.14). Based on our findings of access type and other model parameters, we suggest that, in the case of the 152 journals we analyzed, paying for open access does confer a citation advantage. For authors with limited budgets, we recommend pursuing open access alternatives that do not require paying a fee as they still yielded more citations than closed access. For authors who are considering where to submit their next article, we offer additional suggestions on how to balance exposure *via* citations with publishing costs.

## Introduction

Scientists have a strong interest in ensuring global access to published research. To facilitate this goal, scientific journals have increasingly adopted open access (OA) publishing models. In contrast to traditional subscription-based (or closed access) publishing, OA allows any reader to access articles online without paying a subscription fee. As OA publishing has grown, several OA publishing modalities have emerged, each offering distinct benefits to authors and readers. The “gold” OA category describes articles made freely available at the time of publication directly from the publisher’s website. Further, gold OA articles are explicitly published under an irrevocable open license. While gold OA makes accessing research easiest for readers, it can, in some cases, impose challenges upon authors in the form of an article processing charge (APC). Authors report difficulty in finding funds to cover APCs, with the majority of funding obtained from research grants (American Association for the Advancement of Science, 2022), or a combination of sources, including personal funds (Monaghan, Lucraft & Allin, 2020). APC pricing strategies may limit the number of outlets where they can publish their research or force trade-offs with allocations to other expenditures, such as materials and supplies or conference attendance (American Association for the Advancement of Science, 2022).

Gold OA articles may be published either in open access journals or in traditional, subscription-based journals that give authors the option to pay an APC to make their articles freely available while other articles in the same journal remain behind a paywall. The latter type refers to “hybrid” journals, and this model of publishing articles under an open license in these subscription journals is known as “hybrid gold”. Hybrid gold open access is appealing to authors who wish to publish in well-established journals while simultaneously making their work freely available to non-subscribers. Some of these authors are subject to OA mandates by funding agencies and universities, while others simply want to reduce barriers to prospective readers. The number of subscription-based journals that have introduced a gold OA option has exploded over the past 15 years (Jahn, Matthias & Laakso, 2022; Björk, 2017), which suggests the popularity of hybrid gold OA is growing. During this same period, APCs have risen dramatically. The increase in APCs from 2005–2018 greatly outstripped the rate of inflation in the same time frame, but the volume of articles published in gold open access journals still went up in spite of these fees (Khoo, 2019). Data on year-to-year increases in APCs is scarce due to the difficulty of finding out what various journals charged historically. However, when a small sample of gold open access biology journals for which 2019 APC data were available were checked again in 2022, all but one had increased their APCs, and the number of journals with APCs over $5,000 jumped from one to three (Table 1; Krzton, 2019).

**Table 1:** Gold OA journal APC changes over time. A selection of gold OA biology journals for which 2019 APC data were published (Krzton, 2019) are compared to 2022 APC amounts recorded as part of the present study to illustrate general APC increase over time. All amounts are listed in US currency.

Journal	2019 APC	2022 APC
Genome Biology	3,490	5,030
Nature Communications	5,200	5,790
PLoS Biology	3,000	5,300
Scientific Reports	1,790	2,090
Database: The Journal of Biological Databases and Curation	1,680	2,475
Frontiers in Plant Science	2,950	2,950
Ecology and Evolution	1,950	2,200
PeerJ	1,095	1,395

With increased pressure in recent years to make research open, and rising APCs, authors are left with difficult decisions when choosing how and where to effectively communicate their science (but see below). Due to the relatively recent rise of OA publishing, gold OA journals tend to be younger and lack the long-term brand recognition and marketing power of traditional subscription journals that have introduced OA options. In addition to comparatively higher traditional proxies for prestige (*i.e., Journal Citation Reports* metrics (hereafter, “journal impact”)), hybrid journals often come with higher APCs than fully open journals (Asai, 2022). While traditional considerations of ‘journal impact and target audience remain important for many situations (McKiernan et al., 2019; Chapman et al., 2019; Tregoning, 2018), authors must factor budget into their decisions more heavily now than they did in pre-OA times.

Other OA categories in addition to gold OA have emerged that do not require fees of the authors. Bronze describes articles that are made freely available by the journals themselves and at no cost to the author (Piwowar et al., 2018). However, the process by which articles are selected for bronze designation is unknown and may not be permanent (Piwowar et al., 2018). A perhaps under-utilized alternative to publishing articles OA in either fully OA or hybrid journals is “green” OA, in which authors self-archive their work by uploading preprints to servers like bioRxiv or by depositing post-prints in institutional repositories or other archives (Tennant et al., 2016; Gadd & Troll Covey, 2019). Although green OA is typically subject to journal permissions, formatting restrictions, and embargo periods, there is no cost to the author under this model, making green OA a particularly appealing alternative to costly APCs.

For certain budgets, one of the most important considerations authors face when deciding to pay an APC is whether paying to increase access to their work would translate to increased citations. Thus far, attempts to answer whether OA publishing confers a citation advantage to authors relative to publishing closed access have produced mixed results (Langham-Putrow, Bakker & Riegelman, 2021). While many studies have found support for an OA citation advantage (Emery et al., 2021), others have found the opposite (Dorta-González, González-Betancor & Dorta-González, 2017). Furthermore, studies that have found support for a citation advantage between OA and closed access (Piwowar et al., 2018; Sotudeh, Arabzadeh & Mirzabeigi, 2019; Ottaviani, 2016) have been careful to avoid concluding that OA status leads to greater citations due to methodological and statistical challenges involved in designing a robust citation study that limits the effect of confounders, including language (Lewis, 2018; Basson, Blanckenberg & Prozesky, 2021; Moed, 2007), field or subject area (Archambault et al., 2016; Holmberg et al., 2020; Hubbard, 2017), and journal age. Therefore, any attempt to estimate differences in citation rates between access types must be aware of the potentially confounding forces that may influence citations and account for article attributes that may influence citation rates.

Hybrid journals provide the closest thing to a direct comparison that could be used to test whether a citation advantage for OA publishing exists (Björk, 2017; Harnad & Brody, 2004; Tang, Bever & Yu, 2017). Specifically, hybrid journals circumvent the confounding factor of variation in journal name recognition or perceived quality as both OA and closed access articles can be compared for the same journal. However, few existing studies have taken advantage of the comparison presented by hybrid OA journals to test if OA confers a citation advantage. One such example recovered evidence that OA articles were cited earlier and more frequently than closed access articles published in the same journal during the same period of time (Eysenbach, 2006). However, a comprehensive assessment testing whether OA confers greater citations while taking differeneces among subject areas into account is lacking.

Biology has a higher than average number of hybrid OA papers than other fields (Laakso & Björk, 2016), and a citation advantage for OA has been documented (Archambault et al., 2016; McCabe & Snyder, 2014). Although a few previous studies have looked at the citation pattern in biology, they have largely been limited to just one sub-field within biology (AlRyalat et al., 2019), analyzed relatively small number of records or subset of publications (∼3,500 records; Tang, Bever & Yu, 2017), or a combination of these (Calver & Bradley, 2010; Clements, 2017). Furthermore, the results of these studies are conflicting with regard to whether paying to publish OA actually confers a citation advantage to the authors, with some finding a benefit (Tang, Bever & Yu, 2017; Clements, 2017), and others recovering a minimal effect (Calver & Bradley, 2010). Clements (2017), controlling for self-citation, Journal Impact Factor, number of authors, and article type, investigated the citation patterns and found an OA citation advantage in three marine ecology journals, yet no such advantage was identified in six conservation biology journals (Calver & Bradley, 2010). Due to the limited scope of prior research, it remains unclear whether there is any citations advantage provided by OA across sub-fields in biology.

In this study, we addressed the question of whether authors across sub-fields in the biological sciences can expect to gain more citations by paying an APC to publish OA in a hybrid journal. Using the Clarivate Analytics™ Web of Science Core Collection, we collected a sample of 146,415 articles published in 152 hybrid journals published between 2013 and 2018 to compare the rates of citation between OA and non-OA articles. We used these data to assess (1) the degree to which OA articles published in hybrid journals are cited more than non-OA articles, (2) the contributions of factors such as author count, Journal Impact Factor, and sub-field to citation rates, and (3) if and how these factors influenced any differences in citations rates among access types. Overall, our results show a general citation advantage for OA over closed access, and a clear advantage for hybrid gold OA over other types of OA, but this advantage varies depending on article attributes, such as number of authors or journal metrics. Based on our results, we aim to provide specific and concrete recommendations to authors that should aid decision-making regarding when to and whether it is worthwhile to pay an APC to publish OA in hybrid biology journals.

## Materials and Methods

**Figure 1:**
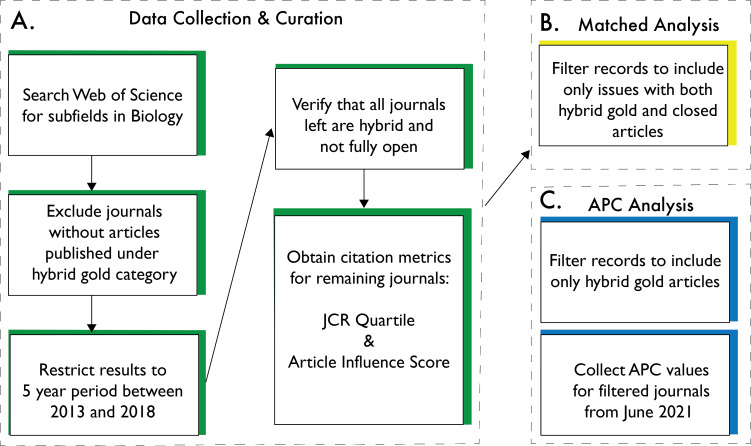
Data preparation. (A) With the permission of the Clarivate Analytics™ legal team, we obtained citation records for articles published in hybrid journals by conducting searches in the Web of Science Core Collection for different Web of Science Categories in biology. We verified whether the journals found by each search were hybrid journals and, if so, we downloaded data, including number of citations, OA type, *etc*., for all articles published in each journal between 2013 and 2018. We also obtained citation metrics that we used as predictors in our full and “matched” analyses. (B) For the matched analysis, we restricted our dataset to compare hybrid gold and closed access articles published in the same volume and issue of our journals. (C) Lastly, we obtained values as of June 2021 for each journal’s APC to test APC values associated with number of citations.

Portions of this text were previously published as part of a preprint (Clark et al., 2022). Our methodology to acquire and curate the data is laid out in Fig. 1. With permission from Clarivate Analytics™, we downloaded a large bibliographic dataset from the Web of Science Core Collection. This project began as a class assignment in which students selected journals based on their subject area of interest. These journals were later classified according to the 12 Web of Science categories encompassing biology: Biochemistry and Molecular Biology, Cell Biology, Entomology, Evolutionary Biology, Genetics and Heredity, Marine and Freshwater Biology, Microbiology, Mycology, Neurosciences and Neurology, Oncology, Plant Sciences, and Zoology. Next, a small cohort of students worked together following the course to re-acquire citation records for the journals identified during the course and to expand the journal list further by adding journals addressing noticeable gaps. Still, the selection of journals was not meant to be exhaustive of all the hybrid journals in each of these categories. Student selected journals were later validated as conforming to a hybrid gold publishing model by excluding all journals that did not include records classified as “Other gold”, the Clarivate Analytics™ Web of Science designation for articles with Creative Commons licenses not published in DOAJ, or solely OA, journals (https://doaj.org/). Our goal was to use the same six-year span for all journals to capture the period in which many subscription-based journals began to offer hybrid publishing options and to also not include articles that had not had enough time to accumulate citations. In our data filtering, we noted some records were included that were outside of the 6-year window. Therefore, we filtered results to remove records not published between 2013 and 2018. We also manually verified whether each journal met the hybrid publishing model requirement (Fig 1). In addition to the bibliographic data obtained for each article (*i.e.,* number of authors and OA status: closed access, bronze, green, or hybrid gold), we collected data for the following journal-level citation metrics from Clarivate Analytics™ Journal Citation Reports (JCR): JCR Quartile within a selected Web of Science category, and Article Influence Score (AIS), which quantifies the average influence of a journal’s article within five years of publication. We also collected APCs as of June 2021 from publisher websites for each journal.

To examine the relationship between OA and citation rates while controlling for other factors, we used generalized linear models. In all models, our response variable was raw citations counts. As citation count is likely non-normally distributed, we initially fit generalized linear models to the data with a Poisson distribution for the response. However, likelihood-ratio (chi-square) tests always indicated that the negative binomial distribution described the data better due to variance inflation in the number of citations (all *χ*^2^ > 3, 437, 870, *p* < 0.0001), and thus we used that distribution for all analyses. In the “full analysis”, we included OA status, author count, JCR quartile (1, 2, 3, or 4), AIS, and year as fixed effects, and field and journal (nested in field) as random effects (Table 2). To improve model convergence and adjust for skewed distributions of the independent variables, we scaled AIS and author count. We also included two-way interaction terms between OA status and each of the other fixed effects. Collinearity among fixed effects was generally low as evidenced by low generalized variance inflation factor scores (all <1.31). Thus, we considered variance inflation not to be an issue in the full model. In all analyses, statistical significance of fixed effects and interactions was assessed *via* Type II Wald chi-square tests using the ‘Anova’ function from the R package ‘car’ v3.1 (Fox & Weisberg, 2022). Pairwise comparisons among groups within a variable were assessed by a Wald Z test with a Bonferroni correction using the ‘emmeans’ v1.8 R package (Lenth et al., 2022). All analyses were done in R (version 4.2.1; R Core Team, 2020) and RStudio (version 2022.07.1+554; RStudio Team, 2020).

**Table 2:** Model parameters for full open access dataset. The various variables included as predictor variables are listed along with random variables in the statistical model of the full dataset. In the “Matched Analysis”, an additional random variable of “volume and issue” was included and the records were subset to only include volumes/issues with both OA and closed access articles. Finally, in the third “APC analysis” model, the predictor variable of APC was added and articles were subset to only include hybrid gold where authors paid an APC, thus removing the predictor of access type. All models shared the same response variable of Citation Counts.

**Predictor variables**	**Response variable**	Response variable
Access type	Field	Citation counts
Author count	Journal in field	
*Journal citation reports* (JCR) quartile		
*JCR* article influence score		
Year		

In a separate analysis, we used a paired design to compare the number of citations between articles published hybrid gold OA and closed access within the same volume and issue of a journal. Thus, we filtered the downloaded records including only issues with both hybrid gold and closed access articles (Fig. 1). These matched data were used in a second statistical analysis (hereafter “matched analysis”) with the response variable of citation count; all independent model parameters for this analysis were the same as those used for the full dataset. However, volume (nested within journal nested within field) and issue (nested within volume nested within journal nested within field) were also included as random effects in the matched analysis. Again, we used a generalized linear model with negative binomial family structure to analyze the data.

In a final sub-analysis, we examined the relationship between citation count and APCs (hereafter “APC analysis”). The full data was restricted to only include articles published under the hybrid gold access model (*i.e.,* those in which APCs had been paid; Fig. 1). A generalized linear model with negative binomial family structure was used to model the relationships between citation count (the response variable) and the same independent variables that were used in the full model analysis with the exception that access type was removed (since all articles were published hybrid gold), and APC charge was included.

## Limitations

There are a few limitations to this study worth noting here. First, the journal selection of 152 is a non-random sample of biology journals from a non-random sample of biology disciplines. To control for this, we included both journal and field in our statistical model as random effects. However, it is worth noting that because we compared green and gold articles from the same journal, any bias in journal selection would only become problematic if that bias were to impact citation rates between articles based on type of access within the same journal. Second, we used 2021 APC rates to analyze the journals due to the difficulty in obtaining past APCs. Recent studies that have accounted for APC changes over time found it to be a labor-intensive data collection step (Butler et al., 2023). Specifically, our approach likely overestimates the actual APCs of the articles analyzed here.

## Results

After filtering, we obtained citation data for 146,415 journal articles from 152 hybrid journals across 12 fields within biology (Table 3). The full list of journals and all raw data are provided *via* an archived GitHub repository (see Data Availability). The number of records per journal averaged 963 and ranged from 15 to 11,286. Records across research fields averaged 12,201 and ranged from 4,575 to 31,253. Across all articles, 61,117 articles were considered closed access whereas 85,298 had some form of OA. Specifically, 18,032 articles were classified as hybrid gold, 9,261 were classified as green, and 58,005 were classified as bronze.

**Table 3:** Breakdown of records by sub-field in Biology. A summary of the data used in this study, including Web of Science category, number of journals targeted and total number of articles used. Additionally, the number of articles in the “matched analysis” are included as well as a break down by access type of each sub-field.

**Research area**	**Number of journals**	**Number of articles**	**Number of matched articles**	**Bronze**	**Closed access**	**Green**	**Hybrid gold**
Biochemistry and Molecular Biology	1	11,286	8,624	28	9,799	969	490
Cell Biology	4	4,575	7	3,868	10	50	647
Entomology	8	5,777	1,567	380	4,669	445	283
Evolutionary Biology	16	16,061	3,007	8,133	4,806	1,186	1,936
Genetics and Heredity	6	7,190	336	2,512	1,016	232	3,430
Marine and Freshwater Biology	6	8,708	2,805	77	7,812	549	270
Microbiology	8	21,921	596	18,700	720	581	1,920
Mycology	16	7,099	1,627	1,059	5,116	346	578
Neurosciences and Neurology	5	6,465	1,366	2,707	1,510	927	1,321
Oncology	5	16,152	1,724	9,250	1,413	898	4,591
Plant Sciences	7	9,928	990	7,005	1,603	144	1,176
Zoology	70	31,253	5,432	4,286	22,643	2,934	1,390
Totals	152	146,415	28,081	58,005	61,117	9,261	18,032

In a simple generalized linear model analyses with access as the only independent variable, we found that hybrid gold articles had an average of 31.1 (30.6–31.5; 95% C.L.) citations, compared to 13.3 (13.2–13.4) citations for closed access articles. Bronze articles averaged 35.9 (35.6–36.2) citations, while green access articles averaged 19.3 (18.9–19.7) citations. All categories of access were statistically different from one another (Wald *z* statistic with Bonferroni correction, all *z* > 16.411, all *p* < 0.0001).

**Figure 2:**
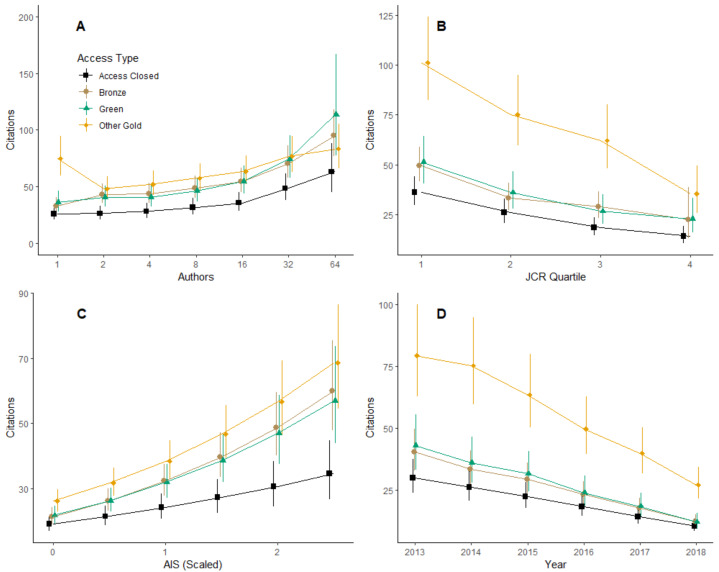
Citations as a function of various model parameters and access type. Access type is color coded to match the named color scheme (*e.g.*, green is indicated in green), except closed access which is indicated in black. (A) Interaction between access type and author count. The number of authors was treated as categorical. Only values under 64 are plotted to emphasize relationships in the majority of the dataset. (B) Interaction between access type and JCR quartile. JCR Quartile of 1 represents the highest journals by Journal Impact Factor. (C) Interaction between access type and scaled (standardized) AIS values. (D) Interaction between access type and year.

Our full model indicated that, in addition to access type, all other variables included in the model had significant relationships with citation counts. Variation in the log number of citations due to the specific journal an article was published in had a standard deviation of 0.27. Similarly, variation in the log number of citations due to the biological sub-field in which an article was published had a standard deviation of 0.15. Moreover, we found that fixed-effects variables all interacted with access type to influence citation counts (Table 4). For example, the model suggested that hybrid gold access generated more citations than the other three access types when articles had few authors, but generated fewer citations than other access types when articles had many authors. Thus, to explore potential non-linearities in the relationship between number of authors and number of citations, as well as the interaction between number of authors and access type, we binned the number of authors into the following discrete categories: 1, 2, 3–4, 5–8, 9–16, 17–32, 33–64, 65–128, 129–256, and >257 authors. However, we note that only 118 (0.08%) articles had more than 64 authors. A likelihood-ratio test indicated that categorizing the author-count variable in this way significantly improved the model (${\chi }_{30}^{2}=467.07$, *p* < 0.0001). Therefore, we used this categorized variable in our full model analysis. The model indicated that with only a single author, hybrid gold generated 2.86 (3.66–2.24; 95% C.L.), 2.25 (2.72–1.86; 95% C.L.), and 2.08 (2.77–1.57; 95% C.L.) times as many citations as closed access, bronze, and green access types respectively (Fig. 2A; all *z* > 6.812, all Bonferroni-adjusted *p* < 0.0001). With only a single author, green and bronze also generated significantly more citations than closed access (*p* = 0.0121 and 0.0061, respectively), but differences were relatively small: 1.38 (1.05–1.81) and 1.27 (1.04–1.54) times as many citations, respectively. With one author, green and bronze access types were not significantly different from each other (*p* = 1.000). This ranked pattern in citations as a function of access was generally maintained between 2 and 32 authors, with hybrid gold generating the most citations, followed by green/bronze, and last by closed access (Fig. 2A). Differences among OA types were not always statistically significant, but OA types always generated significantly more citations than closed access over this author-count range (all *z* > 4.18, all *p* < 0.0002). Above 33 authors, differences among access types were more variable but typically not significantly different (Fig. 2A).

**Table 4:** Results of analysis of full dataset. Type II Wald chi-square tests were used. Thus, significance tests of interactions terms were marginal, but significance tests of main-effects terms were marginal excluding all interaction terms.

Variable	*χ* ^2^	df	*p*-value
Access type	540.16	3	<0.0001
Author count	2,391.91	9	<0.0001
JCR quartile	115.45	3	<0.0001
AIS	109.91	1	<0.0001
Year	27,708.11	5	<0.0001
Access type × Author count	174.90	25	<0.0001
Access type × JCR quartile	64.10	9	<0.0001
Access type × AIS	36.57	3	<0.0001
Access type × Year	141.52	15	<0.0001

Although the full model indicated significant interactions between JCR quartile and access type, as well year of publication and access type (Table 4), the general pattern of hybrid gold > green/bronze > closed access in terms of number of citations held across JCR Quartiles, scaled AIS, and year of publication (Fig. 2). However, the differences among the 4 types of access decreased with higher JCR Quartiles (Fig. 2B), lower scaled AIS scores (Fig. 2C), and year of publication (Fig. 2D). By the 4th quartile, differences between hybrid gold and bronze, and between bronze and closed access were no longer statistically significant (*z* = 1.80, 1.84;  *p* = 0.43 , 0.40, respectively). Similarly, at scaled AIS values <1.5, differences between bronze/green and closed access were not significantly different (all *z* > 2.66, all *p* > 0.05). Finally, from 2016 to 2018, differences between green and closed access were not significantly different (all *z* > 2.56; all *p* > 0.062), and in 2018 differences between bronze and closed access were not significantly different (*z* = 2.02; *p* = 0.26).

Our matched analysis included 28,081 journal articles from 129 journals across the same 12 fields within biology (Table 3). The number of records per journal averaged 218 and ranged from two to 8,624. Records across research fields averaged 2,340 and ranged from seven to 8,624 as some fields were only represented by a single journal. Across all articles, 23,598 were considered closed access whereas 4,483 were classified as hybrid gold.

**Figure 3:**
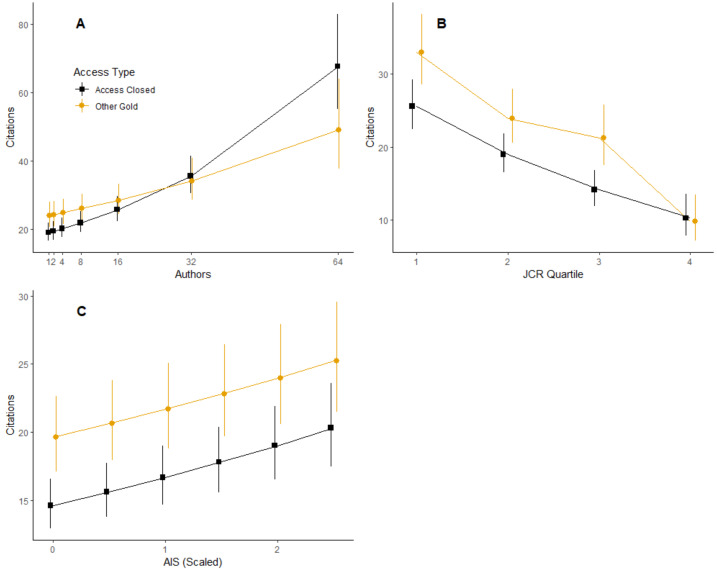
Citations as a function of various variables and access type from same issue of journal. Access type is color coded to match Fig. 2. (A) Interaction between access type and author count. (B) Interaction between access type and JCR quartile. JCR Quartile of 1 represents the highest journals by Journal Impact Factor. (C) Interaction between access type and scaled (standardized) AIS values. Note: Access type by year is not shown here because this interaction term was not significant in this analysis (see Table 5).

Our model of the matched analysis dataset indicated that, in addition to access type, all other variables included in the model had significant relationships with citation counts, but moreover, the variables all interacted with access type to influence citation counts, except for year (Table 5). Unlike with the full dataset, treating author count as a categorical (*i.e.,* binned) variable did not significantly improve the model (${\chi }_{12}^{2}=10.46$, *p* = 0.56). The model indicated that with only a single author, hybrid gold generated 1.26 (1.136–1.4; 95% C.L.) times as many citations as closed access (Fig. 3A; *z* = 4.339, Bonferroni-adjusted *p* < 0.0001). The differences between hybrid gold and closed access decreased with increasing number of authors until 16 authors, at which point differences were not statistically significant (Fig. 3A; *z* = 1.823, Bonferroni-adjusted *p* = 0.068). Above ∼60 authors, differences between access types were once again significant, with closed access generating more citations than hybrid gold (Fig. 3A; *z* =  − 2.290, Bonferroni-adjusted *p* = 0.0220).

**Table 5:** Results of analysis of matched dataset. Type II Wald chi-square tests were used. Thus, significance tests of interactions terms were marginal, but significance tests of main-effects terms were marginal excluding all interaction terms.

Variable	*χ* ^2^	df	*p*-value
Access type	221.06	1	<0.0001
Author count	238.65	1	<0.0001
JCR quartile	86.64	3	<0.0001
AIS	55.73	1	<0.0001
Year	1,846.44	5	<0.0001
Access type × Author count	14.43	1	0.00014
Access type × JCR quartile	19.42	3	<0.0002
Access type × AIS	4.12	1	0.04
Access type × Year	6.86	5	0.23

Similar to the analysis of the full dataset, and despite the interaction, the general pattern of hybrid gold > closed access, in terms of number of citations, held across JCR Quartiles and scaled AIS values (Figs. 3B, 3C. As with the full dataset, the differences between the two types of access decreased with higher-numbered JCR Quartiles (Fig. 3B) or lower scaled AIS scores (Fig. 3C). By the 4th JCR quartile, differences between hybrid gold and closed access were no longer statistically significant (Fig. 3B; *z* =  − 0.407; p = 0 .68). Finally, we observed significantly greater number of citations for hybrid gold compared to closed access at all scaled AIS values, although differences decreased very slightly with increasing AIS values (Fig. 3C; all *z* > 3.677, all *p* > 0.0002).

**Figure 4:**
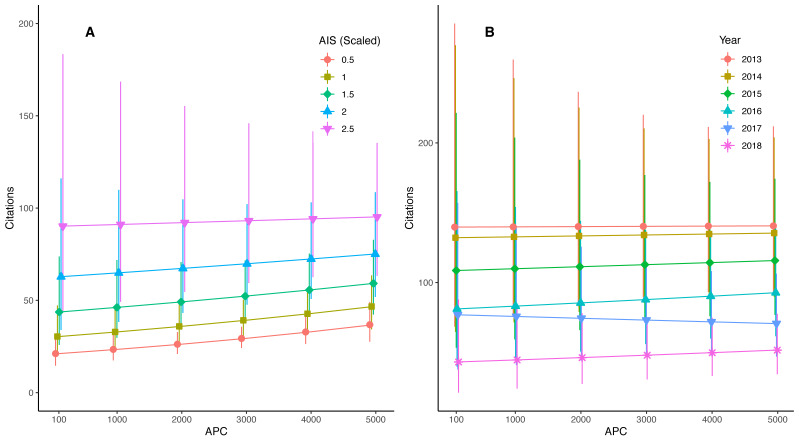
Citations in hybrid gold articles as a function of APC and AIS or year. (A) Interaction between APC and scaled (standardized) AIS values. (B) Interaction between APC and year of publication.

Our APC analysis included 17,542 journal articles from 152 journals across 11 fields; the field of Biochemistry and Cellular Biology was comprised of articles from a single journal, and hence was removed from the analysis due to limited sample sizes that impacted model convergence. The number of records per journal averaged 116 and ranged from one to 2,649. Records across research fields averaged 1,594 and ranged from 270 to 4,501. In a simple analysis of the relationship between number of citations and APCs, we found that for each standard deviation increase in APC (about $1,500), we observed a 19.7% (17.7%–21.9%; 95% C.L.) increase in the number of citations (*p* < 0.0001). However, we also found that for each $1,000 dollar increase in APC, there was about a 1 unit (0.93 ± 0.03; ± 95% C.I.) increase in AIS (standard linear regression; *p* < 0.0001; *r*^2^ = 0.21; note that AIS in the data ranged from 0.17 to 20.8). After statistically controlling for AIS, JCR quartile, author count, and year, we found that the main effect of APC was not significant, but that there were significant interactions between APC and year, as well as APC and scaled AIS values (Table 6). Specifically, increasing APC resulted in slight increases in number of citations at low AIS, but almost no increase in number of citations at high AIS (Fig. 4A). Similarly, increasing APCs resulted in negligible to a slight increase in number of citations for all years except 2017, in which cases increasing APCs resulted in a decrease in the number of citations (Fig. 4B).

**Table 6:** Results of analysis of APC dataset. Type II Wald chi-square tests were used. Thus, significance tests of interactions terms were marginal, but significance tests of main-effects terms were marginal excluding all interaction terms.

Variable	*χ* ^2^	df	*p*-value
APC	2.16	1	0.14
Author count	172.62	1	<0.0001
JCR quartile	35.79	3	<0.0001
AIS	62.51	1	<0.0001
Year	3,284.59	5	<0.0001
APC × Author count	1.91	1	0.17
APC × JCR quartile	5.20	3	0.15
APC × AIS	12.90	1	0.0003
APC × Year	19.50	5	0.0015

## Discussion and Conclusion

In this study we found a significant open access citation advantage for OA articles of all types—gold, green, and bronze—relative to closed access articles published in hybrid journals (Fig. 2). The citation advantage was more pronounced advantage for hybrid gold articles than for bronze or green articles, suggesting that paying an APC to make an article available from the publisher’s website under an open license provides a greater comparative citation advantage (but see further discussion of the APC charges below). However, this pattern may be due to the fact that we operated as if bronze and green articles were made open upon publication, which is not necessarily the case. For example, an article may be considered green if a preprint was made available a year prior to publication, or an article classified as bronze may have only been made open temporarily. Future research investigating whether publishing open access yields more citations should attempt to account for when articles classified as non-gold OA became open and how long they stayed open, using tools such as the Unpaywall^®^ database (Unpaywall, 2024). Two exceptions to this pattern were articles with a large number of authors (> ∼32 authors) and articles published in relatively low profile journals (AIS scores close to 0 and/or in the lowest JCR quartile). We observed the same general patterns when comparing only hybrid gold and closed access articles that were published in the same issue of a journal (Fig. 3).

When publishing under hybrid gold access models, journals with higher AIS scores (*i.e.,* higher citation rate) tend to have higher APCs (Budzinski et al., 2020). Thus, paying the higher APCs associated with high impact hybrid journals may result in more citations, a pattern we recovered support for in our APC analysis. However, after controlling for the effect of journal impact quantified by AIS, higher APCs had minimal effects on citation counts. It is worth noting that our APC values were collected from 2021 despite the records being from 2013–2018. As a result, these do not fully reflect the variation in APCs within journals over time in our dataset. In fact, we found obtaining past values of APCs to be challenging. Therefore, it may be helpful if cost at the time of publishing were included somehow in the meta data of each article through Clarivate Analytics™ to facilitate these types of studies in the future. Disentangling the effects of journal metrics when trying to assess whether paying an APC adds additional citations has proven difficult, as authors may prioritize making their more high profile work open (Craig et al., 2007). Our results were consistent with Piwowar et al. (2018), who found an OA citation advantage primarily driven by hybrid gold publishing.

**Figure 5:**
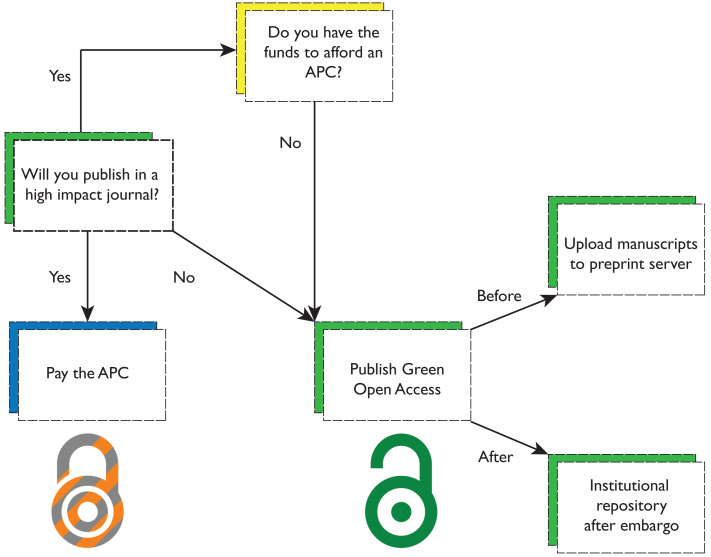
What to consider when publishing open access. Authors have many junctures between article submission and appearance in a journal when they can make their research available while also saving money. After deciding which journal is a good fit for their research, if the journal has a lower Journal Impact Factor, authors can opt for the green route without sacrificing many potential citations (although a low profile journal likely will not have a prohibitively expensive APC). If the journal has a high Journal Impact Factor, whether author(s) should pay an APC comes down to their funding—if they have limited funds, they should forgo paying an APC and opt for the green route as that will be likely to garner more citations than publishing closed access. If authors go with the green route, they can submit their articles to a pre-print server before publication and archive in an institutional repository post-publication; the latter suggestion also applies to articles published closed access older than two years.

Our results indicate that paying an APC for gold OA in a hybrid journal or self-depositing at no cost to the authors is a tradeoff between time and money. Opting for the gold route by paying an APC allows an article to be freely available immediately upon publication, increasing the potential audience size by removing barriers to reader access while the research is new, likely increasing the attention the article receives. Indeed, our results suggest that publishing gold OA article in a hybrid journal maximizes citations in most scenarios. Additionally, choosing to publish gold OA avoids the embargo period imposed by publishers that authors face when choosing to publish green OA, which may last six months or more post-publication. That said, our results do indicate that self-archiving also confers an (albeit less pronounced) citation advantage; therefore, if funds are not available it is still advantageous for authors to deposit their works in repositories (Fig. 5).

Publishers are aware of this tradeoff, as evidenced by the mainstreaming of the hybrid model, the rise of APCs themselves (Budzinski et al., 2020), and increased restrictions on self-archiving (Gadd & Troll Covey, 2019). Though many authors have noticed higher APCs within the same journals over time (Khoo, 2019), historical data on APCs is difficult to find. In 2019, then-current APCs were published for a selection of journals listed in the Directory of Open Access Journals (Krzton, 2019). In a 4-year time frame (between 2019 and 2022), the APCs for all but one journal became more expensive, and one title (PLoS Biology) increased its APC by nearly 77% (Table 1). While these figures are from gold (fully OA) journals rather than hybrid journals, they do reflect the trend of increasing APCs, and several of those journals are controlled by commercial publishers that also have substantial hybrid journal offerings within biology. Along with rising APCs, publishers have increased restrictions on the conditions of self-archiving one’s work, particularly by adding embargoes on green OA deposits (Gadd & Troll Covey, 2019). Beyond putting authors in the difficult position of having to choose between allocating funds to new research or publishing their existing work gold OA, these new barriers threaten to drive further inequity between the Global North and South, which has spurred a growing movement to eliminate APCs altogether (Alperin, 2022; Peterson et al., 2019; Alizon, 2018; Mekonnen et al., 2022).

In light of the foregoing discussion, we offer the following recommendations to authors submitting a manuscript to a biology journal (visualized in Fig. 5):

• **Check whether your institution has a nonexclusive right to deposit prior to publication**. Some universities have adopted policies that assert a nonexclusive right to distribute scholarly work by affiliated personnel *via* the institutional repository on behalf of the authors. These policies are designed to supersede publisher embargoes on self-deposit and may allow for green open access to articles at the time of acceptance without paying an APC.
• **Consider depositing all closed access articles at least two years old**. While it is preferable to know when a publisher will allow a closed access article to be deposited in an open repository prior to submission to a subscription journal, many authors do not realize green OA is an option until after they have a substantial publication history. As green OA articles were found to have a citation advantage in this study and others (Ottaviani, 2016), we suggest authors consider depositing earlier published works when journal policies allow. Embargo periods for accepted manuscripts typically range from six months to two years for self-archiving in an institutional repository, allowing authors to leverage the OA citation advantage for these older articles at no cost to themselves. We recommend contacting scholarly communications units and/or libraries with questions about copyright and self-archiving. Some institutions may even provide assistance with deposit through their scholarly communication units and/or libraries. The online resource Sherpa Romeo (Romeo, 2024) is another helpful tool for finding open access policies.
• **Choice of journal should not be dependent on access status**. Due to the widespread adoption of OA through a variety of channels, most authors today have the option of publishing OA regardless of target journal. Authors should choose the best-fit journal for their research according to their preferred criteria, separate from the issue of when and how to make their work open. The only exception would be if a research sponsor mandates gold OA, which would limit researchers to publishing in gold or hybrid journals.
• **If a research sponsor requires gold OA, their funding should cover the APC.** Authors should review the terms of sponsored research agreements closely to see whether an OA mandate applies to any resulting publications. If sponsors specify immediate public access to the version of record *via* gold OA, authors should request that the sponsor cover the APC if publication fees are not already written into the grant.
• **Authors should save the final accepted manuscript version for later deposit in institutional repositories**. Many journals that permit green OA *via* deposit into an institutional repository still prohibit deposit of the publisher’s PDF with all journal formatting and typesetting applied. When the final version of the manuscript has been approved by the journal editors and all authors, at least one author should retain that version in manuscript form to deposit into an open repository. If the journal requires an embargo period, contact repositories to see whether an immediate deposit is possible with an embargo that will automatically expire on a certain date. This eliminates the need for authors to personally keep track of when they can self-deposit and also minimizes the chances of misplacing the manuscript file in the meantime.
• **Ensure that articles will be published under a recognized open license when paying an APC for gold OA**. When authors pay an APC, it is important to verify that the article will be published under a Creative Commons Attribution (“CC-BY”) or similar open license clearly listed either on the journal page or in the text of the article itself. This guarantees that authors only pay APCs for true gold OA as opposed to temporary free-to-read access.

## Additional Information and Declarations

### Data Availability

The following information was supplied regarding data availability: The data and scripts are available at Zenodo: Amanda Clark, Tanner Myers, Laurie Stevison, steu4718, & Julio Alejandro Yanes. (2022). AUCompBio/OA_2021_AU: v1.0 updated (v1.1). Zenodo. https://doi.org/10.5281/zenodo.7416222. Data included herein are derived from Clarivate Analytics™ Web of Science. Copyright Clarivate 2021.
